# Neutralizing Activity of Vaccine-Induced Antibodies Against Vaccine-Derived Polioviruses Type 2

**DOI:** 10.3390/vaccines14100899

**Published:** 2026-10-14

**Authors:** Evgenia V. Karpova, Elizaveta V. Yakovchuk, Nikita V. Salichkin, Ekaterina A. Korotkova, Olga Y. Baikova, Svetlana E. Sotskova, Alexander P. Ivanov, Olga E. Ivanova, Liubov I. Kozlovskaya

**Affiliations:** 1 Chumakov Federal Center for Research and Development of Immune-and-Biological Products of the Russian Academy of Sciences (Institute of Poliomyelitis) (Chumakov FSC R&D IBP RAS (Institute of Poliomyelitis)), 108819 Moscow, Russia; karpova_ev@chumakovs.su (E.V.K.); yakovchuk_ev@chumakovs.su (E.V.Y.); salichkin_nv@chumakovs.su (N.V.S.); katerkorot@yandex.ru (E.A.K.); baykova_oj@chumakovs.su (O.Y.B.); sotskova_se@chumakovs.su (S.E.S.); ivanov_ap@chumakovs.su (A.P.I.); ivanova_oe@chumakovs.su (O.E.I.); 2 Department of Organization and Technology of Production of Immunobiological Preparations, Institute for Translational Medicine and Biotechnology, First Moscow State Medical University ( Sechenov University), 119048 Moscow, Russia

**Keywords:** VDPV2, seropositivity, poliomyelitis, polio, vaccination, IPV, OPV

## Abstract

Background: Nowadays, poliomyelitis caused by wild polioviruses (PVs) has been virtually eradicated. However, since the removal of the type 2 component from the oral polio vaccine (OPV), immunity to PV2 has declined despite expansion of the use of the inactivated polio vaccine (IPV). Unfortunately, sources of neurovirulent PV2 remain in the world in the form of vaccine-derived poliovirus type 2 (VDPV2) cases and outbreaks and immunodeficient iVDPV2 excretors. Methods: Neutralizing antibodies (nABs) in sera from children with different numbers of IPV/OPV doses were evaluated against VDPV2 strains. Results: A genetic analysis of four VDPV2 strains with varying genetic divergence from Sabin 2 (4.32–17.39%) revealed a number of mutations in antigenic sites. We observed that 6.5–51.8% of undervaccinated (1–2IPV) children did not have nABs against at least one studied VDPV2. Fully vaccinated children showed almost 100% neutralization, although 3.1% vaccinated with 3IPV did not have nABs against three VDPVs; 4.5% vaccinated with 3IPV + 1bOPV and 2.5% vaccinated with 5tOPV did not have nABs against the most divergent iVDPV2 strain. Conclusions: Therefore, some fully vaccinated children may not have detectable levels of protective nABs against VDPV2.

## 1. Introduction

Poliomyelitis is a highly contagious infectious disease caused by the poliovirus, which belongs to the *Enterovirus coxsackiepol* species of the *Picornaviridae* family [1]. There are three different virus serotypes—1, 2, and 3—each of which can cause a wide range of clinical manifestations, from short-term fever with nonspecific symptoms to severe paralytic forms with fatal outcomes or disability. The disease mainly affects children under the age of five and is predominantly transmitted via the fecal–oral route [2]. Nowadays, the disease caused by wild polioviruses has been virtually eradicated thanks to the efforts of the Global Polio Eradication Initiative (GPEI) established by the World Health Assembly in 1988. Since then, the number of poliomyelitis cases has been reduced by 100,000 times, wild poliovirus types 2 and 3 have been eradicated, and only the type 1 wild poliovirus remains endemic only in Afghanistan and Pakistan [3]. Humoral immunity in the form of neutralizing antibodies (nABs, titers over 1:8) is considered the main correlate of protection against paralytic disease [4]. Two different vaccines are currently used for routine immunization of the population worldwide, each with its own mechanism of action and characteristics. The inactivated Salk polio vaccine (IPV) contains three types of killed wild polioviruses and is administered intramuscularly, while the oral polio vaccine (OPV) contains live attenuated Sabin strains and is administered orally [5]. Unlike IPV, OPV not only forms humoral immunity but also creates powerful intestinal immunity that decreases the transmission of poliovirus in subsequent infections [5]. Since attenuated OPV (Sabin) strains replicate in the intestine and are excreted in feces, these viruses can circulate in a non-immune population, leading to the emergence of neurovirulent vaccine-derived polioviruses (VDPVs) [6] and cause outbreaks of poliomyelitis [7]. VDPVs are classified according to the number of nucleotide substitutions in the VP1 capsid protein [8]. Moreover, depending on the isolation origin, they are divided into three main groups: (1) circulating vaccine-derived polioviruses (cVDPVs), resulting from prolonged uncontrolled circulation of the vaccine virus among populations with low immunization coverage; (2) vaccine-derived polioviruses occurring in immunocompromised individuals (iVDPVs), which are isolated from people with primary immunodeficiencies; and (3) ambiguous vaccine-derived polioviruses (aVDPVs), which have genetically evolved but whose source has not been identified [7]. In recent years, cVDPV2 has been responsible for the vast majority of all polio cases worldwide [9]. To address this problem, and in relation with the eradication of wild poliovirus type 2, a global strategic transition in 2016 was undertaken: the trivalent oral vaccine (tOPV) containing all three types of the virus was replaced with a bivalent vaccine (bOPV) containing only types 1 and 3 [5,10]. To prevent the population from losing immunity to type 2 poliovirus, it was recommended that at least one dose of IPV be administered as part of routine immunization [11,12]. This measure known as the “switch” has failed, as the number of outbreaks associated with cVDPV2 increased sharply, and one dose of IPV did not provide sufficient immunity against PV2 [13]. Considering this, the WHO has recommended the introduction of at least two doses of IPV for routine immunization [5]. However, the removal of the type 2 component from OPV created a new vulnerability: the level of population immunity to type 2 poliovirus began to decline, especially in regions where it is difficult to ensure high IPV coverage [14], which at any moment could lead to major poliomyelitis outbreaks. Therefore, the Strategic Advisory Group of Experts on Immunization (SAGE) now recommends a vaccination schedule in which a child should receive three doses of IPV in its first year of life [15]. Despite all the aforementioned efforts, access to IPV is restricted in several countries, and global coverage with two IPV doses in 2025 was 68% [16]. Moreover, due to IPV shortage after the tOPV–bOPV switch in 2016, several countries received vaccination against PV2 with a delay [17], and in small amounts that did not cover the entire population at risk. On the other hand, routine vaccination schedules recommend three doses administered at one-month intervals, followed by a fourth dose approximately one year after the third [18]. This leaves a window for infection.

Therefore, changes in immunization recommendations and low overall IPV coverage coexist in the world with a continued presence of neurovirulent PV2 sources, including VDPV2 cases, even outbreaks, and iVDPV2 excreters. This study aimed to evaluate the neutralizing activity of blood sera from children vaccinated under different immunization regimens against VDPV2 strains.

## 2. Materials and Methods

### 2.1. Ethical Statement

This study was approved by the Ethical Committee of Chumakov FSC R&D IBP RAS (Institute of poliomyelitis) protocol No. 25052026-2 on 25 May 2026.

### 2.2. Poliomyelitis Vaccination Schedule Used in Russia

Over the past 20 years, the poliomyelitis immunization schedule in Russia has changed. Until 2007, only trivalent OPV was used; the immunization schedule consisted of 6 vaccine administrations to children at the ages of 3, 4.5, 6, 18, and 20 months, and 14 years. Since 2008, a sequential immunization schedule has been used. From 2008 to 2010, the schedule included 3 IPV doses for children at the ages of 3, 4.5, and 6 months, followed by trivalent OPV (bOPV since April 2016). In the period 2011–2021, the number of IPV doses was reduced to two. Since 2022, the immunization schedule consists of 4 IPV doses at the ages of 3, 4.5, 6, and 18 months, and 2 doses of bOPV at the ages of 20 months and 6 years. Children from certain groups (those suffering from primary immunodeficiency conditions, children from orphanages) have received only IPV since 2008.

### 2.3. Serum Samples

Serum samples were collected in September–December 2019 from children aged 1–15 years as part of the Pilot Project to assess population immunity against poliomyelitis in the Russian Federation according to order of Russian Federal Service for Surveillance on Consumer Rights Protection and Human Wellbeing (Rospotrebnadzor) No. 346, dated 10 June 2019; all sampled children were vaccinated according to the Russian national immunization schedule. Exclusion criteria for the serum collection were immunodeficiency (primary, immunosuppressive therapy, HIV infection), acute infection, and contraindications for blood collection. For the study, 251 serum samples with known vaccination data were selected (dates of vaccinations and vaccine series are presented in Table S1). The sera were collected from children vaccinated with 1 to 4 doses of IPV, 2IPV + 2bOPV or 3IPV + 1bOPV, and 5tOPV (Table 1). Serum samples were stored at −20 °C before use.

### 2.4. Poliovirus Strains

Four VDPV2 strains isolated during poliomyelitis surveillance in Russia in 2015–2023 were amplified in HEp2c cells. Each virus was then titrated in HEp2c cells and titers were expressed as the 50% cell culture infectious dose (CCID_50_) [19]. In addition to the VDPV2 isolates, the vaccine strain Sabin 2, used in OPV, and the wild poliovirus type 2 strain MEF, used in IPV, from the laboratory collection were utilized.

### 2.5. Neutralization Test

The neutralization test was performed in HEp2c (Cincinnatti) cells (obtained from MHRA, London, UK) according to the standard protocol [20]. Briefly, serial two-fold dilutions of serum samples were prepared in 96-well plates in 2 × EMEM medium (Chumakov FSC R&D IBP RAS (Institute of Poliomyelitis), Moscow, Russia), and then mixed with equal volumes of viral suspension, containing approximately 100–300 CCID_50_ per well to obtain a final dilution series of 1:8–1:1024. After incubation for 3 h at 36.5 °C, a suspension of cells (approximately 1 × 10^4^ cells per well) in 2 × EMEM medium containing 5% FBS (Invitrogen, Waltham, MA, USA) was added to the wells. Each experiment included titration of the virus dose in the inoculum to ensure an acceptable dose range; negative cell culture control and standard positive serum with known nAB titer (prepared according to [20]). After 5 days of incubation, signs of a cytopathic effect (cell death) were visually assessed using light microscopy. The titer of nABs was calculated using the Karber method. Sera that did not inhibit the cytopathic effect at a minimum dilution of 1:8 (log_2_ titer < 3) were considered negative. All serum samples from one group were tested against a particular virus simultaneously in one experiment.

### 2.6. VDPV2 Genome Sequencing

Viral RNA was extracted from an infected cell suspension using RIBOPrep kit (AmpliSence, Moscow, Russia), reverse-transcribed with random hexamer primers using SuperScript III Reverse Transcriptase (Invitrogen, Waltham, MA, USA), and amplified with PCR with different specific primers (primer sequences are available up on request) using DreamTaq DNA Polymerase (Thermo Scientific, Waltham, MA, USA). PCR fragments were purified via gel electrophoresis and extracted using the QIAquick Gel Extraction kit (Qiagen, Hilden, Germany). Sequencing was performed on an ABI PRISM 3100 Genetic Analyzer using BigDye Terminator v3.1 Cycle Sequencing Kit (Thermo Scientific, Waltham, MA, USA).

Sequences were analyzed with Lasergene software (DNASTAR, Madison, WI, USA).

### 2.7. Statistical Analysis

Statistical analysis and data visualization were performed using OriginPro 8 software (OriginLab Corp., Northampton, MA, USA). The geometric mean titer (GMT) and standard deviation (SD) were calculated for serum titers. Percentages and 99% confidence intervals (CIs) were calculated for negative-sample ratios. Statistical tests are presented in Section 3.

## 3. Results

### 3.1. Genetic Description of VDPV2 Strains

Three diverged VDPV2 strains were selected from laboratory collection for the present work: 53344 [21], 74146, and 76174 were isolated from sewage in different locations in Russia in 2015–2023, and the cVDPV2 strain 67270 isolated from a healthy migrant from Tajikistan during the outbreak in 2020–2021 [22].

The cVDPV 67270 genome was the least diverged from Sabin 2 and contained 4.31% mutation in the VP1 sequence (Table 2). Sewage isolated strains were more divergent: 76174 had 7.31% mutations in VP1, 74146 had 12.18%, and 53344 had 17.39% (Table 2).

**Figure 1:**
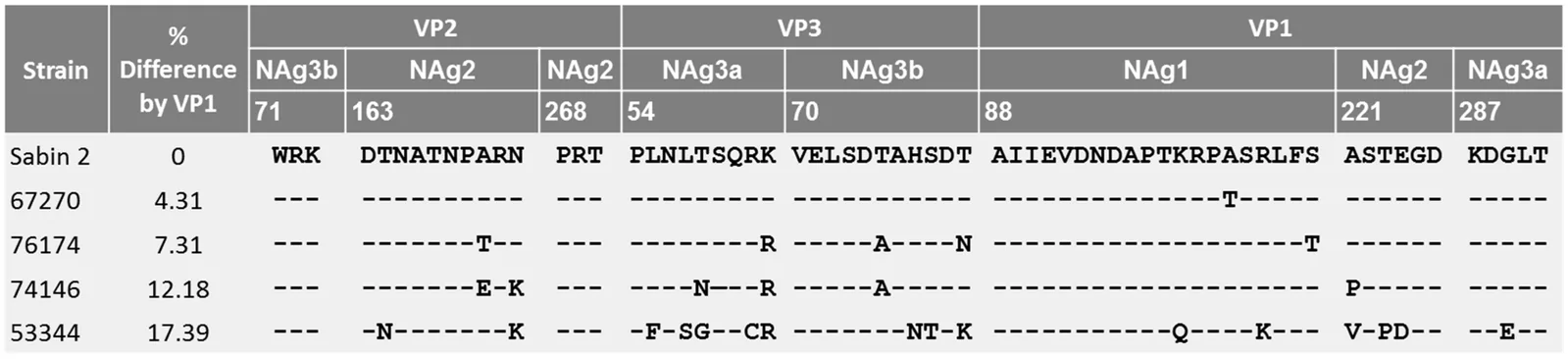
Mutations in antigenic sites of the capsid proteins VP1–VP3 of the studied VDPV2 strains.

Analysis of the ratio of amino acid identity to nucleotide identity of the VP1 sequence (Table 2) and genetic patterns of elevated amino acid substations accumulation (Figure 1) [23] suggested that the most divergent strains 53344 and 74146 were most likely iVDPVs, while the less mutated strain 76174 occupies a position between iVDPVs and cVDPVs, mostly due the small number of mutations from Sabin (Table 2).

As antigenic properties of the poliovirus capsids can be affected by mutations in antigenic sites (NAg) [24,25,26], we analyzed VP1–VP3 capsid protein sequences of the studied VDPV2 (Figure 1). The cVDPV2 strain 67270 had only one mutation in NAg1. The highest number of substitutions were present in antigenic sites in the most divergent strain 53344, with changes occurring throughout NAg1–3. The antigenic sites of strains 74146 and 76174 contained fewer substitutions. These mutations in the antigenic sites of possible iVDPVs 53344, 74146, and 76174 can potentially help these viruses to escape neutralization by polio-vaccine-induced antibodies.

### 3.2. Seropositivity Rates to VDPV2 Strains

We evaluated the neutralizing activity of 251 blood serum samples against all studied VDPV2 strains with varying genetic divergences from the vaccine strain Sabin 2, OPV strain Sabin 2, and IPV strain MEF. Serum samples with nAB titers ≥ 1:8 were considered positive.

Sera were collected from children with different vaccination histories: 1 to 4 doses of IPV, 2IPV + 2bOPV or 3IPV + 1bOPV, and 5tOPV. Each group of sera included 22–80 samples (Table 1).

**Figure 2:**
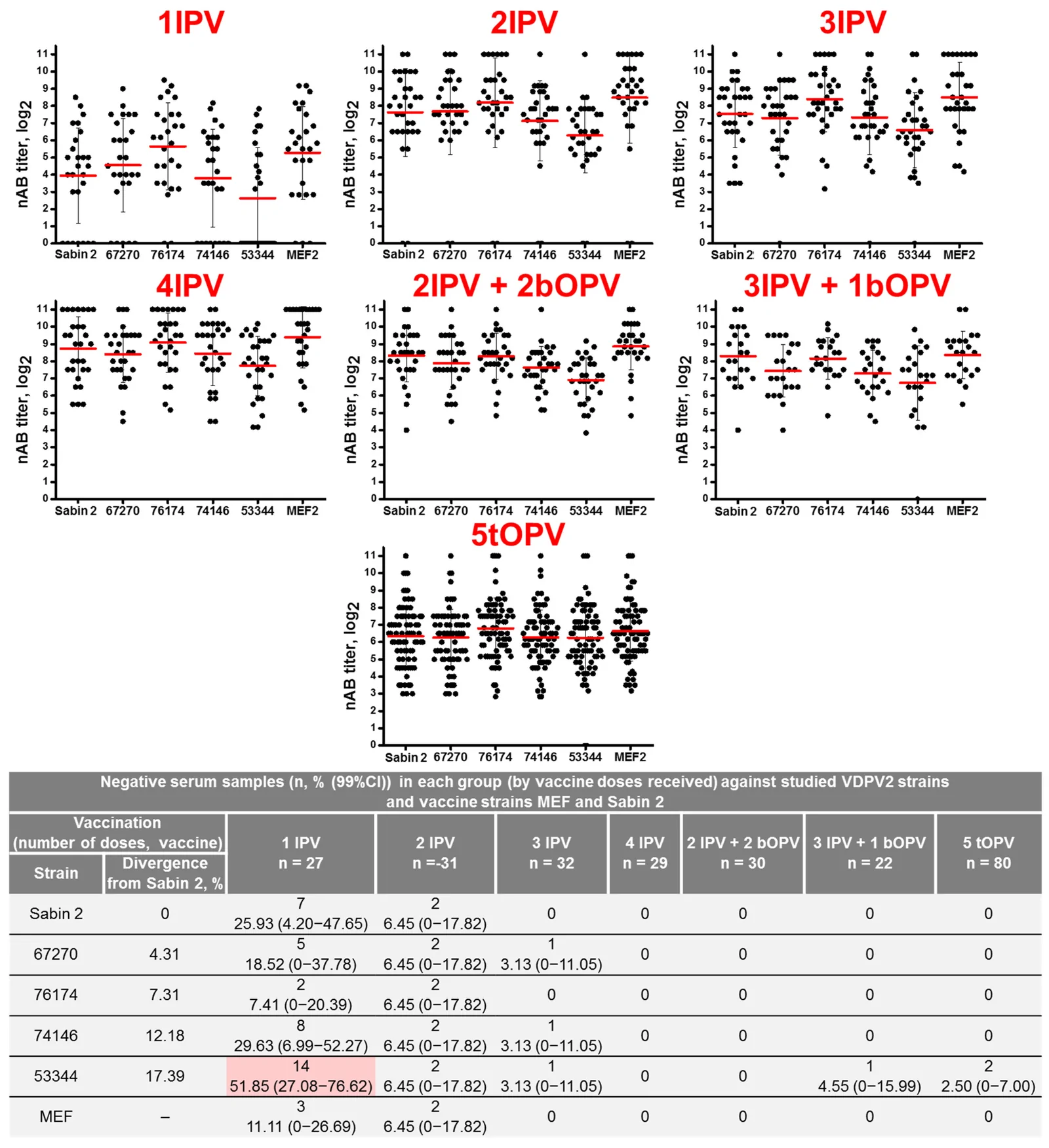
Neutralizing antibody titers to VDPV2 strains, vaccine strain Sabin 2, and wild poliovirus type 2 strain MEF in sera of vaccinated children with 1–4 doses of IPV; by combination schedule with 2 IPV + 2 bOPV, 3 IPV + 1 bOPV, or with 5 tOPV. Serum samples with nAB titers ≥ 1:8 were considered positive; serum samples without detected nAB were considered 0 in calculations. Neutralizing antibody (nAB) titers of individual serum are presented as dots; geometric mean titers (GMT) are presented as red horizontal lines; whiskers represent SD of GMT. Differences in nAB titers against each strain in each group of serum samples are statistically significant (*p* < 0.05, Friedman ANOVA). Negative-sample numbers (*n*, %, 99%CI) are presented as a table. The seropositivity rate (%) in the 1IPV vaccinated group against VDPV2 53344 highlighted in red differ significantly from the groups vaccinated with different regimens (*p* < 0.05, Chi-square with Bonferroni correction). IPV—inactivated poliovaccine based on wild poliovirus strains (including type 2 strain MEF); OPV—oral life poliovaccine based on attenuated Sabin strains (including type 2 strain Sabin 2).

Overall, the seropositivity rates to the vaccine strains (IPV strain MEF and OPV strain Sabin 2) increased with the increase in the number of doses received. Sera collected from children vaccinated with one or two IPV doses showed rates under 100% (Figure 2).

Overall, nAB titers against VDPV2 strains in each group of serum samples were significantly lower than nAB titers to IPV strain MEF (*p* < 0.05, Friedman ANOVA), except for the tOPV vaccinated children (no significance for comparison with vaccine strains Sabin 2 or MEF). Moreover, the difference was more prominent with the increase in the divergence from Sabin 2 (Figure 2): nAB titers against strains 74146 (12.18% divergence) and 53344 (17.39% divergence) showed the greatest differences. However, titers of the cVDPV2 strain 67270 (4.31% divergence) were lower than titers of the more mutated VDPV2 strain 76174 (7.31% divergence) (Figure 2).

Sera collected from children vaccinated with 1IPV showed 78.9% seroconversion to IPV strain MEF. From 7.4% to 51.9% of serum samples (Figure 2) did not contain nABs against Sabin 2 or studied VDPV2 with the lowest seropositivity rates to the most diverged strain 53344. Seropositivity rates (%) against VDPV2 strain 53344 for this group of serum samples were significantly lower than in other groups of serum samples (*p* < 0.05, Chi-square with Bonferroni correction).

Sera collected from children vaccinated with 2IPV showed 93.5% seroconversion to IPV strain MEF; 6.5% of the same serum samples did not contain nABs against Sabin 2 or studied VDPV2.

Serum samples from children vaccinated with 3–4IPV, 5OPV or a combined schedule 2IPV + 2OPV or 3IPV + 1OPV showed 100% seropositivity rates to vaccine strains Sabin 2 and MEF. Almost 100% seropositivity rates to all studied VDPV2 with some exception; one serum sample (3.1%) from a child received 3IPV did not contain nABs against cVDPV2 67270 and possible iVDPV2, 76146, and 53344 (Figure 2). One serum sample (4.5%) from a child that received 3IPV + 1bOPV and two samples (2.5%) from children that received 5tOPV when the last dose was given 9 and 11 years prior did not exhibit detectable nABs against the most divergent VDPV2 strain 53344 (Figure 2).

It should be noted that negative serum samples had the same characteristics, i.e., dates of vaccination and time between the last vaccination and serum collection, as the other samples of the group (Table 1 and Table S2).

## 4. Discussions

Three doses of Salk-IPV are considered a full primary vaccination course that forms protective immunity against polio. For countries using OPV, a combination schedule including at least two IPV doses and three or more bOPV doses is recommended [15]. However, average seroconversion rates to PV2 after Salk-IPV are as follows: 1 dose—41%, 2 doses—71–89% [5], and 3 doses—93–99% [27]. Therefore, a small portion of vaccinees do not have nABs, even after complete vaccination schedule of three IPV doses. Moreover, gaps in global humoral immunity to polioviruses type 2 can be attributed to several factors: (1) restricted access to IPV, resulting in routine immunization schedules with only 2IPV in several countries [16,17,18]; (2) an IPV shortage after the tOPV–bOPV switch [17], leading to a delay in IPV implementation in several countries; and (3) routine vaccination schedules recommending a fourth dose to be delivered approximately one year after the third one [18].

In recent years, cVDPV2 has been the most widespread poliovirus [13,28,29], posing a worldwide threat, including even in polio-free countries [30,31,32,33]. Furthermore, the problem of long-term poliovirus excreters possessing a threat for the polio-free world has long been anticipated [34,35,36]. Summarizing the above, since the tOPV–bOPV switch in 2016, VDPV2 poses the greatest threat to the world.

The mutation accumulation patterns of cVDPV2 and iVDPV2 differ [23], and thus, the same percent of divergence can indicate mutation accumulation in different parts of the genome that can differently affect the virus neutralization by the ABs produced by vaccinees’ immune systems. On the other hand, the spectrum of nABs induced by vaccination with different schedules differ as well. The studied VDPV strains differed in the pattern of amino acid substitutions accumulation in the antigenic sites: possible iVDPVs 76174, 74146, and 53344 contained multiple mutations in all antigenic sites of VP1–VP3 proteins, whilst cVDPV2 67270 contained only one conservative mutation, i.e., Ala to Thr in the VP1 protein. Therefore, the studied VDPVs can possibly escape mutants, avoiding neutralization with vaccine-induced antibodies, although the data does not directly support this suggestion.

We have evaluated the seropositivity rates against VDPV2 strains with different genetic divergences from vaccine strain Sabin 2 in sera of children vaccinated with 1–4IPV, 5tOPV, and IPV and bOPV in combination.

Seroconversion levels to vaccine strains Sabin 2 and MEF reached 100% in sera from children with three or more polio vaccine doses (3–4IPV, 5tOPV, 2IPV + 2bOPV, 3IPV + 1bOPV), which is to be expected [5]. Sera from undervaccinated children (1–2IPV) showed 91.4% seroconversion levels.

Level of nABs against the least diverged cVDPV2 67270 associated with the recent outbreak in Tajikistan were detected in sera of fully vaccinated children (three or more vaccine doses), with one exception of a child vaccinated with 3IPV showing low nAB titers to all studied viruses, including the IPV strain MEF. Sera from undervaccinated children (1–2IPV) in 6.5–18.5% of cases did not contain nABs against 67270. The same serum samples did not contain nABs against divergent 74146 and 53344 VDPVs. Therefore, the presence of serum samples of 1–3IPV-vaccinated children without detectable levels of antibodies against real-world cVDPV2 and iVDPVs suggests the possible susceptibility of such children for poliovirus infection and provided the basis recommendation of four or more IPV doses for routine vaccination against poliomyelitis.

Several countries use only two IPV doses for routine immunization [18]; thus, not only seroconversion to PV type 2 is below 90% [5] but also some vaccinated children may not have nABs against VDPVs, which creates conditions for VDPV2 circulation and outbreak.

Levels of nABs against the most mutated VDPV2 53344 were detected in most sera from fully vaccinated children (three or more vaccine doses comprising 3–4IPV, 5tOPV, 2IPV + 2bOPV, or 3IPV + 1bOPV). Negative samples belonged to children vaccinated with 3IPV + 1bOPV or 5tOPV. These samples showed low nAB titers relative to other studied viruses, including Sabin 2 and MEF. People vaccinated with only tOPV comprise a large part of the world population. From our results, we can expect that some of them do not have detectable levels of nABs against divergent VDPVs with antigenic patterns such as 53344.

The main limitation of this study is the comparatively small number of tested serum samples. However, the presence of samples from fully vaccinated children (3IPV, 5tOPV, 3IPV + 1bOPV) that did not contain detectable levels nABs against at least one of the studied VDPV2 shows a potential lack of protection in such individuals against divergent VDPV2. One can argue that an nAB booster during the first 24 h after infection or possible effects of non-neutralizing antibodies would raise the protective level of immunity, but we were unable to evaluate this. Moreover, the sera were collected from children several months after the last vaccination and adolescents tOPV-vaccinated 10 years prior, which signifies the peak immunity levels [5]. No antibody response (or a weak response) may be due to immune deficiencies in the individual. However, in our case, this factor can be ruled out, as such donors were excluded during serum collection.

The current SAGE recommendation of three or more IPV doses for routine immunization [15] should induce high enough humoral immunity levels to protect vaccinees against paralytic disease caused by genetically diverged VDPV2. However, to strengthen the immunity levels, four or more IPV dose vaccination schedules should be recommended and maintained, even in a polio-free world.

## 5. Conclusions

We conclude that children vaccinated with 3IPV may not have detectable levels of nABs against cVDPV2 and divergent VDPV2. Some children that are fully vaccinated using a combined schedule (3IPV + 1bOPV) or only tOPV (five doses) may not have detectable levels of nABs against highly divergent VDPV2. Therefore, these raise questions about the protection of such children from poliomyelitis.

## Figures and Tables

**Table 1:** Classification of sera by polio vaccine doses received by the child.

Vaccine and Number of Doses	Years of Birth	Age at the Time of the Blood Collection Me [Q1;Q3], Years	Last Vaccination Year (Mean Date)	Time from the Last Vaccination to the Blood Collection, Me [Q1;Q3], Weeks	Number of Samples
1IPV	2017–2018	2 [1;2]	2018–2019 (11 January 2019)	43 [10;71]	27
2IPV	2016–2018	2 [1;2]	2018–2019 (19 April 2019)	37 [8;50]	31
3IPV	2016–2018	2 [1.5;2]	2018–2019 (30 January 2019)	44.5 [32;57.5]	32
4IPV	2012–2017	2.5 [2;4]	2015–2019 (03 January 2019)	30.5 [21;41]	29
2IPV + 2bOPV	2016–2018	2 [2;2]	2018–2019 (16 May 2019)	23 [19;36]	30
3IPV + 1bOPV	2009–2018	2 [2;4]	2011–2019 (03 May 2018)	24.5 [17;66]	22
5tOPV	2003–2008	13 [12;13.5]	2005–2010 (03 November 2008)	571 [537;607.5]	80

**Table 2:** Description of VDPV2 strains analyzed in this study.

VDPV2	Isolation Site	Isolation Source	Year	No. of nt Mutations/VP1	% nt Mutations/VP1	% nt Identity/VP1	% aa Identity/VP1	% nt Mutations/P1
67270	Voronezh	Healthy	2021	39	4.32	95.7	98.0	96.8
76174	Dagestan	Sewage	2023	66	7.31	92.6	96.3	92.2
74146	Tver	Sewage	2023	110	12.18	87.6	96.0	88.4
53344	Moscow	Sewage	2015	157	17.39	81.2	90.7	82.4

## Data Availability

All data supporting the results is presented in the text and Supplementary Materials.

## Supplementary Materials

The following supporting information can be downloaded at: https://www.mdpi.com/article/10.3390/vaccines14100899/s1: Table S1. Neutralization test tiers; Table S2. Description of serum samples with negative results in the neutralization test against the studied VDPV2, Sabin 1, and MEF.
