# Supplementary material for: Neutralizing Activity of Vaccine-Induced Antibodies Against Vaccine-Derived Polioviruses Type 2
Source: Vaccines (Basel). 2026 Oct 14;14(10):899. doi: 10.3390/vaccines14100899 (PMC13709473; doi:10.3390/vaccines14100899)
Supplement: Supplementary file 1 [file vaccines-14-00899-s001.zip › Table S2.pdf]

Statistical analysis data

Table S2. Description of serum samples with negative results in neutralization test against studied VDPV2, Sabin 1 and MEF.

Vaccination	N	Sabin2		67270		76174		74146		53344		MEF2		Remark
		N	%	N	%	N	%	N	%	N	%	N	%	
1 IPV	27	7	25.9	5	18.5	2	7.4	8	29.6	14	51.8	3	11.1	approx. MEF neg. were neg. against other viruses, last vaccination, 2018-2019
2 IPV	31	2	6.5	2	6.5	2	6.5	2	6.5	2	6.5	2	6.5	same samples, last vaccination, IPV, 2018 and 2019
3 IPV	32			1	3.1			1	3.1	1	3.1			the same sample, last vaccination, IPV, 2018
4 IPV	29													
2 IPV + 2 bOPV	30													
3 IPV + 1 bOPV	22									1	4.5			last vaccination, IPV, 2019
5 tOPV	80									2	2.5			last vaccination, tOPV, 2008 and 2010
